# Efficacy of everolimus with exemestane versus exemestane alone in Asian patients with HER2-negative, hormone-receptor-positive breast cancer in BOLERO-2

**DOI:** 10.1007/s12282-013-0444-8

**Published:** 2013-02-13

**Authors:** Shinzaburo Noguchi, Norikazu Masuda, Hiroji Iwata, Hirofumi Mukai, Jun Horiguchi, Puttisak Puttawibul, Vichien Srimuninnimit, Yutaka Tokuda, Katsumasa Kuroi, Hirotaka Iwase, Hideo Inaji, Shozo Ohsumi, Woo-Chul Noh, Takahiro Nakayama, Shinji Ohno, Yoshiaki Rai, Byeong-Woo Park, Ashok Panneerselvam, Mona El-Hashimy, Tetiana Taran, Tarek Sahmoud, Yoshinori Ito

**Affiliations:** 1 Department of Breast and Endocrine Surgery, Osaka University, Osaka, Japan; 2 Department of Surgery, Breast Oncology, National Hospital Organization, Osaka National Hospital, Osaka, Japan; 3 Department of Breast Oncology, Aichi Cancer Center Hospital, Nagoya, Japan; 4 Department of Breast Oncology, National Cancer Center Hospital East, Kashiwa, Japan; 5 Breast and Endocrine Surgery, Gunma University Hospital, Maebashi, Japan; 6 Songklanagarind Hospital, Faculty of Medicine, Prince of Songkla University, Songkhla, Thailand; 7 Siriraj Hospital, Mahidol University, Bangkok, Thailand; 8 Department of Breast and Endocrine Surgery, Tokai University Hospital, Isehara, Japan; 9 Department of Surgery, Tokyo Metropolitan Cancer and Infectious Diseases Center Komagome Hospital, Tokyo, Japan; 10 Department of Breast and Endocrine Surgery, Kumamoto University, Kumamoto, Japan; 11 Department of Breast and Endocrine Surgery, Osaka Medical Center for Cancer and Cardiovascular Diseases, Osaka, Japan; 12 Department of Breast Oncology, NHO Shikoku Cancer Center, Matsuyama, Japan; 13 Department of Surgery, Korea Cancer Center Hospital, Seoul, Korea; 14 Department of Breast Oncology, National Kyushu Cancer Center, Fukuoka, Japan; 15 Department of Breast Surgery, Hakuaikai Sagara Hospital, Kagoshima, Japan; 16 Department of Surgery, Yonsei University College of Medicine, Seoul, Korea; 17 Department of Global Oncology Development, Novartis Pharmaceuticals Corporation, East Hanover, NJ USA; 18 Department of Medical Oncology, Breast Oncology Center, Cancer Institute Hospital, Japanese Foundation for Cancer Research, Tokyo, Japan

**Keywords:** Advanced breast cancer, Endocrine resistance, Everolimus, Exemestane, Progression-free survival

## Abstract

**Background:**

The addition of mTOR inhibitor everolimus (EVE) to exemestane (EXE) was evaluated in an international, phase 3 study (BOLERO-2) in patients with hormone-receptor-positive (HR^+^) breast cancer refractory to letrozole or anastrozole. The safety and efficacy of anticancer treatments may be influenced by ethnicity (Sekine et al. in Br J Cancer 99:1757–62, 2008). Safety and efficacy results from Asian versus non-Asian patients in BOLERO-2 are reported.

**Methods:**

Patients were randomized (2:1) to 10 mg/day EVE + EXE or placebo (PBO) + EXE. Primary endpoint was progression-free survival (PFS). Secondary endpoints included overall survival, response rate, clinical benefit rate, and safety.

**Results:**

Of 143 Asian patients, 98 received EVE + EXE and 45 received PBO + EXE. Treatment with EVE + EXE significantly improved median PFS versus PBO + EXE among Asian patients by 38 % (HR = 0.62; 95 % CI, 0.41–0.94). Median PFS was also improved among non-Asian patients by 59 % (HR = 0.41; 95 % CI, 0.33–0.50). Median PFS duration among EVE-treated Asian patients was 8.48 versus 4.14 months for PBO + EXE, and 7.33 versus 2.83 months, respectively, in non-Asian patients. The most common grade 3/4 adverse events (stomatitis, anemia, elevated liver enzymes, hyperglycemia, and dyspnea) occurred at similar frequencies in Asian and non-Asian patients. Grade 1/2 interstitial lung disease occurred more frequently in Asian patients. Quality of life was similar between treatment arms in Asian patients.

**Conclusion:**

Adding EVE to EXE provided substantial clinical benefit in both Asian and non-Asian patients with similar safety profiles. This combination represents an improvement in the management of postmenopausal women with HR^+^/HER2^−^ advanced breast cancer progressing on nonsteroidal aromatase inhibitors, regardless of ethnicity.

## Introduction

Worldwide, breast cancer is the most common malignancy in women and one of the leading causes of cancer deaths [1–3]. Incidence of breast cancer in Asia is increasing [3]. In Asia, as in Western countries, treatment approaches for breast cancer typically follow National Comprehensive Cancer Network [4] and St. Gallen guidelines. For postmenopausal patients with hormone-receptor-positive (HR^+^) advanced breast cancer, aromatase inhibitors (steroidal or nonsteroidal) are the standard initial treatment [4]. Even so, most patients are unresponsive to initial treatment or acquire resistance. Other treatment options include estrogen receptor (ER) antagonists (e.g., tamoxifen) and ER downregulators (e.g., fulvestrant). These treatment options provide limited clinical benefit once endocrine resistance develops (especially after aromatase inhibitor therapy), and survival is poor [5]. New treatment options that can offer patients with advanced breast cancer the hope of overcoming resistance and that can prolong the time of effectiveness of endocrine therapy and delay chemotherapy are needed.

Hyperactivation of the mammalian target of rapamycin (mTOR) pathway is associated with breast cancer progression and with the development of endocrine resistance [6]. Aberrations in phosphatidylinositol 3-kinase (PI3K)/mTOR pathway protein expression are also associated with poor prognosis in HR^+^ breast cancer [7]. However, in vitro and in vivo data indicate that mTOR inhibitors can inhibit cell proliferation and restore sensitivity to fulvestrant, letrozole, and tamoxifen [8–11].

Everolimus (Afinitor^®^, Novartis) is an orally active mTOR inhibitor. It is approved for the treatment of patients with progressive neuroendocrine tumors of pancreatic origin, advanced renal cell carcinoma, and subependymal giant cell astrocytoma associated with tuberous sclerosis [12]. Recently, everolimus (EVE) was also approved in combination with exemestane (EXE) for use in the USA and the 27 European Union member states, plus Iceland and Norway, and in Mexico, Argentina, and other Latin American countries, for the treatment of postmenopausal patients with HR^+^ breast cancer whose disease has progressed during or after nonsteroidal aromatase inhibitor therapy. This approval was based on outcomes from BOLERO-2. In this phase 3 study, EVE + EXE improved progression-free survival (PFS) compared with EXE + placebo (PBO; median PFS = 7.8 months vs 3.2 months, respectively; hazard ratio [HR] = 0.45; *P* < 0.0001) [12].

Variations in the pharmacodynamics and pharmacokinetics of anticancer agents can be attributed in part to ethnic differences, potentially resulting in alterations of their safety and efficacy profiles [13]. In fact, some studies of targeted therapies have shown that variability in safety and efficacy is associated with patient ethnicity [14]. To ensure an optimal treatment response is balanced with a manageable safety profile, the potential inter-ethnic differences in anticancer drug effects should be considered [13]. Treatment for lung cancer using the epidermal growth factor inhibitor gefitinib, for example, is more effective in Asian patients than in patients of other ethnicities [15]. The incidence of interstitial lung disease (ILD) is also more prevalent in Asian patients treated with gefitinib monotherapy than in those of other ethnicities [15]. ILD is one of the relatively common, serious adverse events (AEs) associated with molecular targeted anticancer therapies, and treatment with EVE has been associated with ILD [16, 17]. Thus, it is important to compare the frequency of AEs, including ILD, induced by EVE in both Asian and non-Asian patients.

To determine whether patient ethnicity has an effect on the efficacy and safety of EVE + EXE, we performed an analysis in Asian versus non-Asian patients with HR^+^ advanced breast cancer in BOLERO-2 after a median follow-up of 18 months.

## Patients and methods

The BOLERO-2 study is an international, phase 3, multicenter, randomized, double-blind, placebo-controlled trial (ClinicalTrials.gov identifier NCT00863655). The protocol and results for the entire study have been reported [16, 18]. Post hoc analyses of the data from Asian patients (who selected Asian as their race at randomization) and non-Asian patients included in BOLERO-2 are reported herein.

### Patients

Patients were postmenopausal women with metastatic or locally advanced, estrogen receptor-positive (ER^+^) human epidermal growth factor receptor-2 nonamplified (HER2^−^) breast cancer that had recurred or progressed during or after letrozole or anastrozole therapy as described previously [16]. This study was conducted in accordance with the Declaration of Helsinki, in agreement with the institutional review board at each participating center, in accordance with Good Clinical Practice and applicable local regulations. Every patient provided written informed consent.

### Study design

Patients were randomized (2:1) to EVE (10 mg/day) + EXE (25 mg/day) or PBO + EXE (25 mg/day). Randomization was stratified according to sensitivity to endocrine therapy and the presence of visceral metastasis. Treatment continued until disease progression, intolerable toxicity, or withdrawal of consent. During the study, dose reductions or interruptions were allowed to manage AEs. Crossover from the PBO arm to the EVE arm was not allowed.

### Study endpoints

The primary endpoint was PFS, defined as the time from randomization to the first documentation of disease progression (as assessed by the local investigator according to Response Evaluation Criteria in Solid Tumors [RECIST] [19] or, in the case of nonmeasurable disease, unequivocal progression or appearance of new lesions) or death from any cause. The key secondary endpoint was overall survival. Other secondary endpoints included overall response rate (ORR), clinical benefit rate (CBR), and time to overall response and duration of overall response according to RECIST [19].

### Efficacy and safety assessments

An independent data monitoring committee (IDMC) was responsible for monitoring safety and pharmacokinetic data as well as reviewing efficacy results at the interim and final analyses. Tumor evaluation based on computed tomography or magnetic resonance imaging was performed at baseline (within 6 weeks before randomization) and every 6 weeks thereafter until disease progression and initiation of further anticancer therapy. Objective tumor response and disease progression were assessed per RECIST version 1.0 [19]. AEs were assessed at each study visit and were graded according to the National Cancer Institute Common Terminology Criteria for Adverse Events, version 3.0 [20].

### Patient-reported outcomes

Quality of life (QOL) was evaluated using the European Organization for Research and Treatment of Cancer Quality of Life Questionnaire-Core 30 (EORTC QLQ-C30; Version 3.0, 2001), a reliable and valid questionnaire developed to assess the quality of life of cancer patients [21, 22]. This self-administered questionnaire is composed of 30 items arranged into a number of functional and symptom subscales as well as a global health status (GHS)/global QOL subscale, which was the primary QOL variable of interest for BOLERO-2.

### Statistical analyses

Progression-free survival was based on the intent-to-treat analysis, according to the randomized treatment group and stratification. Distribution of PFS was estimated using the Kaplan–Meier method, and the HRs and corresponding 95 % confidence intervals (CIs) were estimated using the Cox proportional hazard model. In addition, the protocol-specified time to definitive deterioration (TTD) in the EORTC QLQ-C30 GHS score (defined as a 5 % decrease in QOL relative to baseline, with no subsequent increase above this threshold) was calculated in the Asian subset using Kaplan–Meier estimates and was described using medians and 95 % CIs. The TTD was compared between EVE + EXE and PBO + EXE using a log-rank test.

## Results

### Patient characteristics

**Fig. 1:**
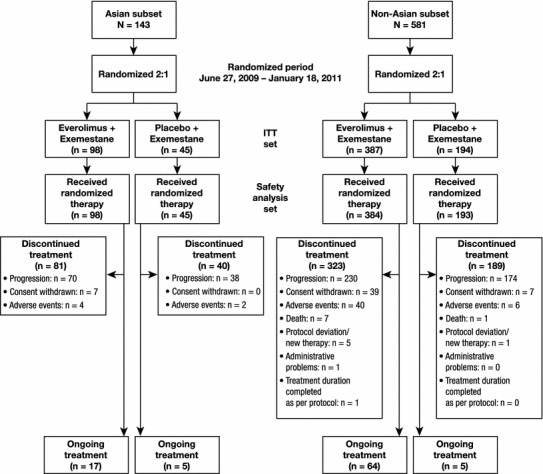
CONSORT flowchart. *ITT* intention-to-treat. Ongoing treatment refers to those patients at time of cutoff for this analysis. Note that disease progression events in this figure are those that resulted in treatment discontinuation

Median follow-up was 18 months at the time of this analysis (cutoff date 15 December 2011). Of the 724 patients in BOLERO-2, 143 were Asian, with 106 (74.1 %) of Japanese origin. There were 98 Asian patients in the EVE + EXE arm and 45 in the PBO + EXE arm (Fig. 1).

Patient and disease characteristics at baseline among the Asian and non-Asian patients were generally comparable, although the Asian patients were younger and a greater proportion had good performance status (Table 1). Among the Asian population, there were more patients in the EVE + EXE arm who had at least 3 sites of metastases compared with the PBO + EXE arm. In the PBO + EXE arm, Asian patients had less visceral disease than non-Asian patients. Prior treatments at study entry were mostly similar between Asian and non-Asian patients. However, more non-Asian patients in the EVE + EXE arm received chemotherapy in the metastatic setting than Asian patients (Table 1).

**Table 1:** Demographics of Asian versus Non-Asian population

Baseline demographics	Asian		Non-Asian	
	Everolimus + exemestane (*n* = 98)	Placebo + exemestane (*n* = 45)	Everolimus + exemestane (*n* = 387)	Placebo + exemestane (*n* = 194)
Age, years				
Mean (SD)	59.9 (7.2)	58.6 (8.2)	63.1 (10.9)	61.8 (10.0)
Median (range)	59.5 (40.0–79.0)	60.0 (28.0–72.0)	63.0 (34.0–93.0)	61.0 (38.0–90.0)
Age group, %				
<65 years	77.6	82.2	55.3	62.9
≥65 years	22.4	17.8	44.7	37.1
Ethnicity, %				
Chinese	5.1	0	0	0
Japanese	72.4	77.8	0	0
Mixed	1.0	0	2.1	3.1
Hispanic/Latino	0	0	7.2	5.2
Indian (subcontinent)	0	0	0.3	0
Other	21.4	22.2	90.4	91.8
Number of metastatic sites, %^a^				
1	33.7	33.3	31.5	25.3
2	22.4	33.3	32.6	35.6
≥3	42.8	33.3	35.7	39.2
ECOG performance status, %				
0	82.7	86.7	54.8	53.1
1	15.3	13.3	41.1	40.2
2	0	0	2.3	3.6
Time between initial diagnosis and 1st recurrence/metastasis, %				
<3 months	13.3	11.1	22.2	20.1
3 to <6 months	0	0	1.3	2.6
≥6 months	80.6	80.0	69.3	71.6
Missing	6.1	8.9	7.2	5.7
Metastatic cancer sites, %				
CNS^b^	2.0	0	1.0	0
Visceral (excluding CNS)^c^	59.2	53.3	58.1	60.8
Lung	34.7	31.1	28.2	33.5
Liver	31.6	22.2	33.9	32.5
Lung and liver	9.2	4.4	9.0	12.4
Bone	69.4	51.1	78.3	83.5
Bone only	20.4	11.1	22.0	21.1
Other	56.1	73.3	49.1	53.6
Previous chemotherapy, %				
Adjuvant/neoadjuvant only	60.2	48.9	39.5	37.6
Metastatic only	6.1	11.1	15.8	9.3
Both	10.2	15.6	12.4	16.0
Number of previous chemotherapy lines in advanced setting, %				
1	16.3	26.7	28.2	23.7
2	0	0	0	0

Data from 15 December 2011 safety update cutpoint

*CNS* central nervous system, *ECOG* Eastern Cooperative Oncology Group, *SD* standard deviation

^a^One patient each in the Asian and non-Asian subgroups had missing information

^b^CNS includes spinal cord, brain and meninges

^c^Visceral includes lung, liver, pleural, pleural effusions, peritoneum, and ascites

The median durations of exposure to treatment were longer in Asian patients than in non-Asian patients. Among Asian patients, median exposure to EVE was 27.6 weeks, whereas median exposure to EXE was 32.6 weeks in the EVE + EXE arm and 18.0 weeks in the PBO + EXE arm. Among non-Asian patients, median exposure to EVE was 23.7 weeks; median exposure to EXE was 28.1 weeks in the EVE + EXE arm and 13.9 weeks in the PBO + EXE arm (Table 2).

**Table 2:** Duration of exposure to study treatment

	Asian patients				Non-Asian patients			
	Everolimus + exemestane (*n* = 98)		Placebo + exemestane (*n* = 45)		Everolimus + exemestane (*n* = 384)		Placebo + exemestane (*n* = 193)	
	Everolimus	Exemestane	Placebo	Exemestane	Everolimus	Exemestane	Placebo	Exemestane
Duration (weeks)								
Median	27.6	32.6	18.0	18.0	23.7	28.1	13.1	13.9
Range	2.0–123.3	2.0–123.3	2.0–101.0	4.0–101.0	1.0–109.4	1.0–109.4	1.0–82.0	1.0–82.0

The percentages of patients who required EVE dose reductions or interruptions were similar between the Asian and non-Asian patients (71.4 vs. 65.6 %), as were the percentages of Asian and non-Asian patients who required EXE dose reductions or interruptions while receiving EVE + EXE (22.4 vs. 24.2 %), respectively. In contrast, Asian patients receiving PBO + EXE required more EXE dose reductions or interruptions than non-Asian patients (26.7 vs. 8.3 %), respectively. Most of these dose reductions or interruptions were the result of an AE (data not shown). At the time of cutoff, 15.4 % of Asian patients and 11.9 % of non-Asian patients were ongoing with study treatment (Fig. 1). Among Asian patients, 82.7 % discontinued EVE + EXE treatment and 88.9 % discontinued PBO + EXE treatment, whereas 83.5 and 97.4 % of non-Asian patients discontinued EVE + EXE and PBO + EXE treatment, respectively (Fig. 1). Most of the patients who discontinued treatment did so because of disease progression.

### Efficacy

**Fig. 2:**
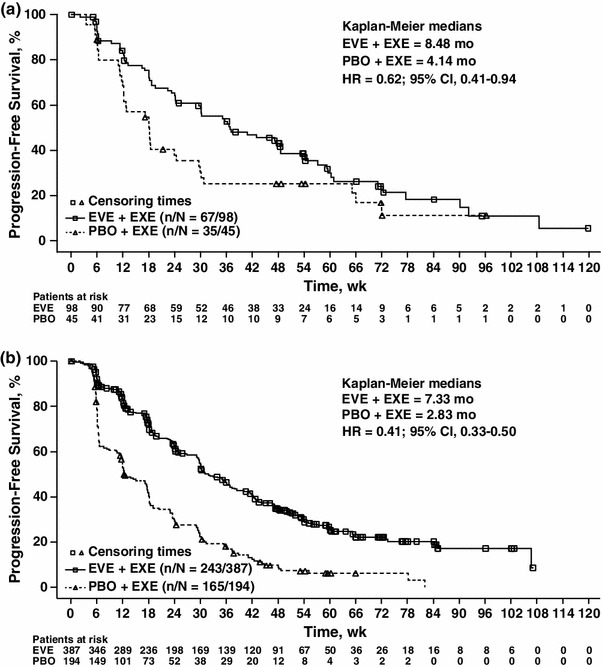
Kaplan–Meier analyses of progression-free survival in **a** Asian and **b** non-Asian patients with advanced breast cancer. *CI* confidence interval, *EVE* everolimus, *EXE* exemestane, *HR* hazard ratio, *PBO* placebo

The combination of EVE and EXE reduced the risk of disease progression by 38 % among Asian patients compared with PBO + EXE (HR = 0.62; 95 % CI, 0.41–0.94; Fig. 2). At the cutoff date, 17.3 % of Asian patients in the EVE + EXE arm and 11.1 % of patients in the PBO + EXE arm were progression free and remained on treatment, whereas 71.4 % of Asian patients in the EVE + EXE arm and 84.4 % of patients in the PBO + EXE arm had disease progression (Fig. 1). Median PFS per local investigator assessment among Asian patients in BOLERO-2 was 8.48 months for EVE + EXE versus 4.14 months for PBO + EXE (Fig. 2).

**Fig. 3:**
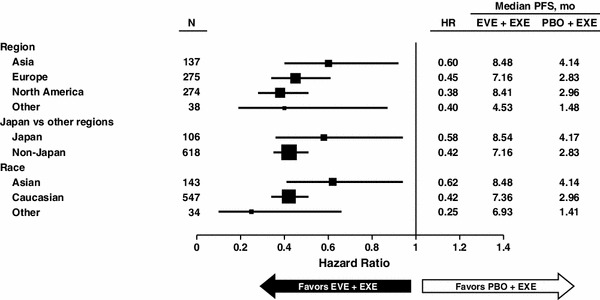
Forest plot of progression-free survival subgroup analysis by region and ethnicity. Subsets were prespecified in the analysis plan. Data from 18-months’ median follow-up. *EVE* everolimus, *EXE* exemestane, *HR* hazard ratio, *PBO* placebo, *PFS* progression-free survival

Japanese patients comprised the largest subset within the Asian subgroup, and nearly 15 % of the overall BOLERO-2 patient population. Therefore, additional analyses specific to the Japanese subset were feasible, and indicated that treatment with EVE + EXE significantly improved median PFS versus PBO + EXE by 42 % (HR = 0.58) in these patients. The median PFS results also favored the combination of everolimus and exemestane in European and North American patients (Fig. 3).

There were no complete responses (CRs) recorded for either the EVE + EXE or the PBO + EXE arm. No partial responses (PRs) were observed with PBO + EXE in the Asian subset, compared with 19 PRs (19.4 %) in the EVE + EXE arm based on local investigator assessment. Overall, Asian patients had greater CBR and ORR in the EVE + EXE arm than in the PBO + EXE arm (CBR, 58.2 vs. 28.9 %; ORR, 19.4 % vs. 0, respectively; Table 3).

**Table 3:** Best response

	Asian		Non-Asian	
	Everolimus + exemestane (*n* = 98)	Placebo + exemestane (*n* = 45)	Everolimus + exemestane (*n* = 387)	Placebo + exemestane (*n* = 194)
Best overall response, %				
Complete	0	0	<1	0
Partial	19	0	10	2
Stable disease	66	78	73	55
Progressive disease	11	20	10	36
Unknown	3	2	7	8
Objective response rate, %^a^	19	0	11	2
Clinical benefit rate, %^b^	58	29	50	26

^a^Complete and partial responses

^b^Complete and partial responses plus stable disease ≥24 weeks

For non-Asian patients, the median PFS per investigator assessment in the 2 arms was 7.33 months and 2.83 months, respectively (HR = 0.41; 95 % CI, 0.33–0.50; Table 3, Fig. 2). Based on local investigator assessment, there were 3 CRs and 39 PRs (10.1 %) among non-Asian patients in the EVE + EXE arm versus no CRs and 4 PRs in the PBO + EXE arm. The CBR and ORR for non-Asian patients were 49.6 and 10.9 % in the combination arm versus 25.8 and 2.1 % in the PBO + EXE arm, respectively (Table 3).

### Safety

Across the entire study, the most common treatment-emergent AEs in the EVE + EXE arm included stomatitis and rash; these were also the most common AEs among both Asian and non-Asian patients (Table 4) [15]. Some AEs were reported in a higher percentage of Asian patients compared with the non-Asian patients. These included stomatitis, rash, dysgeusia, pneumonitis, nail disorder, increased LDH, nasopharyngitis, and ILD. Specifically, the rates of grade 1 and 2 dysgeusia were higher in Asian versus non-Asian patients in the EVE + EXE arm (30.6 vs. 19.8 %) but comparable in the PBO + EXE arm (6.7 vs. 5.7 %). The incidence of nasopharyngitis was similar across treatment arms, but much higher in Asian than non-Asian patients in both the EVE + EXE (22.4 vs. 7.0 %) and PBO + EXE (20.0 vs. 6.2 %) arms; all events were grades 1 or 2 (Table 4). Pneumonitis, which was reported only in the EVE + EXE arm, was higher in Asian patients than in non-Asian patients in the EVE + EXE arm (23.5 vs. 14.1 %, respectively). However, the frequency of grade 3 and 4 pneumonitis was lower in Asian patients compared with non-Asian patients (2.0 vs. 3.6 %, respectively). In contrast, hot flushes were comparable in incidence between Asian and non-Asian patients. They were, however, less frequent in Asian and non-Asian patients in the EVE + EXE arm (6.1 and 5.5 %) than in the PBO + EXE arm (13.3 and 14.5 %) (Table 4).

**Table 4:**
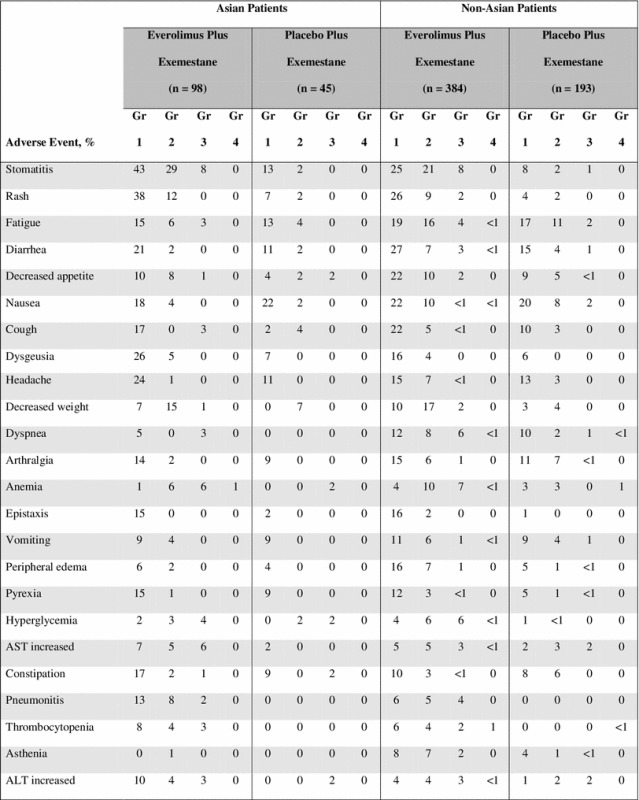

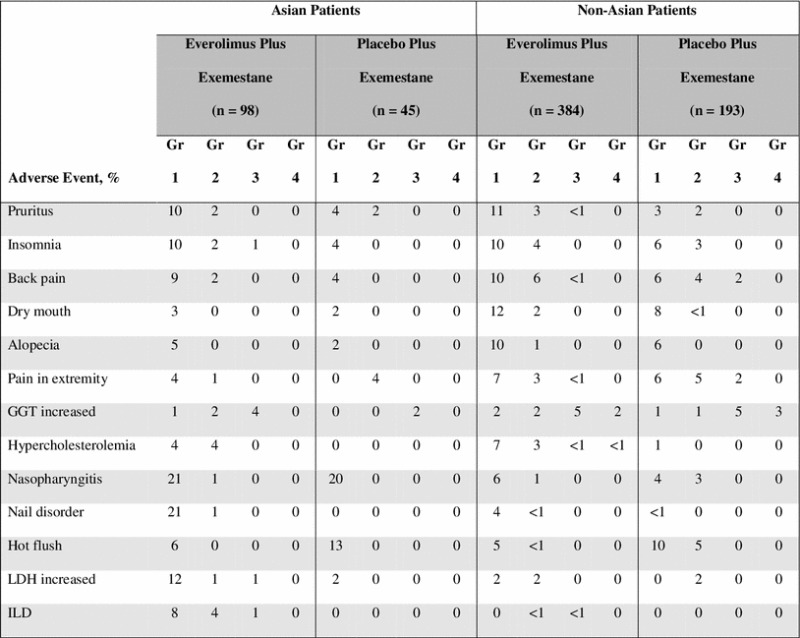
Treatment-emergent adverse events with at least 10 % incidence in the everolimus + exemestane arm in the Asian and non-Asian subpopulations

*ALT* alanine aminotransferase, *AST* aspartate aminotransferase, *GGT* gamma-glutamyltransferase, *ILD* interstitial lung disease, *LDH* lactate dehydrogenase

Notably, the incidence of grade 3 and 4 AEs among patients who received EVE + EXE was generally similar or lower in Asian patients compared with non-Asian patients (Table 4). The only exceptions were increased aspartate aminotransferase (AST) levels and cough. The most common grade 3 and 4 AEs (≥5 %) for both Asian and non-Asian patients in the EVE + EXE treatment group included stomatitis (8.2 vs. 7.8 %), anemia (7.1 vs. 7.6 %), increased AST levels (6.1 vs. 2.9 %), hyperglycemia (4.1 vs. 6.0 %), and dyspnea (3.1 vs. 5.7 %), respectively. There were very few grade 4 AEs reported, regardless of treatment arm or ethnicity subset, and none were reported in at least 5 % of the patients studied (Table 4).

### Quality of life in Asian patients

**Fig. 4:**
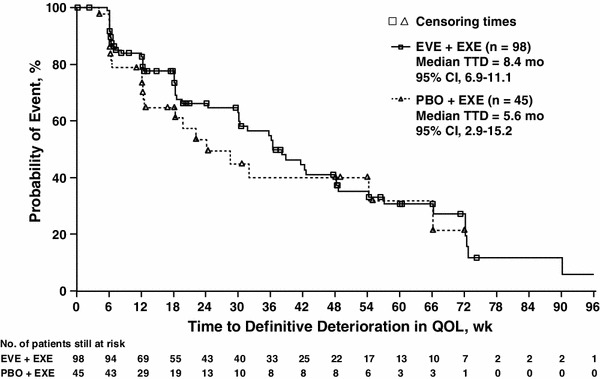
Time to deterioration in EORTC QLQ-C30 (5 % decrease from baseline). *CI* confidence interval, *EORTC QLQ-C30* European Organization for Research and Treatment of Cancer Quality of Life Questionnaire-Core 30, *EVE* everolimus, *EXE* exemestane, *PBO* placebo, *TTD* time to definitive deterioration

Treatment with EVE + EXE did not affect TTD in EORTC QLQ-C30 GHS compared with PBO + EXE in Asian patients. At the protocol-defined threshold of 5 % decrease from baseline, the median TTD was 8.4 months (95 % CI, 6.9–11.1 months) in the EVE + EXE arm compared with 5.6 months (95 % CI, 2.9–15.2 months) in the PBO + EXE arm (HR = 0.79; 97.5 % CI, 0.44–1.44; Fig. 4).

## Discussion

Although women with HR^+^ breast cancer often respond to multiple lines of endocrine therapy, most ultimately progress. When patients with HR^+^ advanced breast cancer progress despite nonsteroidal aromatase inhibitors, the current treatment paradigm includes EXE followed by tamoxifen, toremifene, or fulvestrant [4]. This paradigm is followed in Asia as well as in Western countries. Once patients progress on initial endocrine therapy, the available treatment options offer limited clinical benefit and poor survival [5]. New treatment options are needed that can offer patients with advanced breast cancer the hope of overcoming resistance, prolong the time for which endocrine therapy is effective, and delay chemotherapy.

In the phase 3 BOLERO-2 study, the addition of EVE to EXE increased median PFS by 4.6 months [12]. These results suggest that inhibition of cross-talk pathways (PI3K/mTOR) may help improve outcomes in this patient population. Nearly 20 % of the 724 patients in this study were Asian, providing an opportunity to determine the efficacy and safety of EVE in this important subgroup.

Ethnic differences can account for variations in both the pharmacokinetics and pharmacodynamics of anticancer agents, potentially resulting in alterations of the safety and efficacy profiles of these agents [13]. For example, docetaxel, like gefitinib [15], has demonstrated enhanced efficacy in Asian versus Caucasian patients [13]. This was accompanied, however, by higher incidence of febrile neutropenia requiring hospitalization [13]. CYP2D6 genetic polymorphisms have been shown to affect the conversion of tamoxifen to its most active metabolite, endoxifen. As a result, the efficacy of tamoxifen might vary according to the distribution of these genetic polymorphisms among various ethnic populations [13]. The distribution of genetic polymorphisms affecting CYP2D6 activity is different between Asian and non-Asian patients. Thus, it is hypothesized that the efficacy of tamoxifen may also be different between these patient populations [13]. To ensure optimal treatment response and understand the safety profile, it is important to consider the potential inter-ethnic differences in anticancer drug effects [13].

We have demonstrated in this report that the efficacy of EVE is consistent between the Asian and non-Asian subgroups. Combining EVE with EXE more than doubled the median PFS versus EXE with PBO, from 4.14 to 8.48 months for Asians and from 2.83 to 7.33 months for non-Asians. Asian patients also experienced a greater CBR and ORR after receiving EVE + EXE versus PBO + EXE. Median exposure to EVE + EXE was nearly 4 weeks longer in Asian versus non-Asian patients. Despite the longer exposure to EVE + EXE in Asian patients, the frequency of drug discontinuation for these patients was lower than for non-Asian patients. Also, there were no significant differences in TTD of EORTC QLQ-C30 GHS for the Asian subset of patients. Finally, treatment-emergent AEs were comparable across the two groups.

Some AEs (e.g., stomatitis, nasopharyngitis, pneumonitis, and ILD) were slightly more frequently reported among Asian patients. Others (e.g., anemia) were less frequent. However, all AEs were generally consistent with those reported for EVE in the overall BOLERO-2 study [16]. Similar AEs were seen in other indications following EVE treatment [23]. Occurrences of grade 3 and 4 anemia, stomatitis, abnormal liver enzymes, fatigue, and hyperglycemia have also been frequently reported in Japanese patients with metastatic gastric cancer treated with EVE monotherapy following progression on chemotherapy [24]. Effective management of AEs associated with the use of EVE requires patient education, physician awareness, and early intervention [16]. In some cases (e.g., more severe instances or higher grades of these AEs), dose modifications and standard care have proven useful [12, 25].

Interstitial lung disease (ILD; characterized by the inflammation of the interstitium of the lung) and non-infectious pneumonitis (characterized by the presence of non-infectious, nonmalignant infiltrates) are known side effects of mTOR inhibitors [26, 27]. In the current study, AEs including ILD and non-infectious pneumonitis were coded using the MedDRA terminology (version 14.0) and were assessed as described in the “Methods” section. An increased frequency of ILD has been reported in Japanese cancer patients receiving molecular targeted anticancer therapies such as gefitinib and erlotinib [15]. The patterns of ILD were also the focus of a recent study that retrospectively evaluated 7 Japanese patients treated with EVE for advanced renal cell carcinoma [28]. Patients with mild ILD were able to continue EVE treatment. More severe ILD led to EVE discontinuation and short-term steroid therapy, which generally resulted in rapid resolution of ILD. Prompt recognition of ILD incidence or exacerbation, and exclusion of progressive disease or infection, were determined to be of paramount importance for the successful management of these AEs [28]. The frequency of ILD overall was higher in the Asian patients in this BOLERO-2 study; nonetheless, grade 3 and 4 ILD occurred with similar low frequencies in Asian and non-Asian patients. Whereas pneumonitis, like ILD, was more prevalent in Asian patients treated with EVE + EXE, some of the symptoms of pneumonitis, such as dyspnea and cough, were less frequent in the Asian patients in the EVE + EXE arm. This demonstrates that EVE treatment is not associated with any exacerbated safety concerns based on patient ethnicity.

In conclusion, combining EVE with EXE provided substantial clinical benefit to both Asian and non-Asian patients. EVE was well tolerated and most of the EVE-related AEs were manageable. Observed AEs in BOLERO-2 were consistent with AEs previously reported for rapamycin analogues [29, 30]. This combination of EVE + EXE did not affect self-assessed QOL in Asian patients. Thus, EVE + EXE represents an important improvement in the management of postmenopausal women with HR^+^ HER2^−^ advanced breast cancer progressing after nonsteroidal aromatase inhibitor treatment, regardless of ethnicity.
